# The economic burden of oral cancer in Iran

**DOI:** 10.1371/journal.pone.0203059

**Published:** 2018-09-27

**Authors:** Aziz Rezapour, Reza Jahangiri, Alireza Olyaeemanesh, Bita Kalaghchi, Mojtaba Nouhi, Azin Nahvijou

**Affiliations:** 1 Health Management and Economics Research Center, Iran University of Medical Sciences, Tehran, Iran; 2 Department of Health Economics, School of Health Management and Information Sciences, Iran University of Medical Sciences, Tehran, Iran; 3 Health Equity Research Center, Tehran university of medical sciences, Tehran, Iran; 4 National Institute for Health Research, Tehran University of Medical Sciences, Tehran, Iran; 5 Radiation oncology research center, Tehran university of medical sciences, Tehran, Iran; 6 Cancer Research Center of Cancer Institute, Tehran University of Medical Sciences, Tehran, Iran; Sciensano, BELGIUM

## Abstract

**Background:**

Cancer is one of the leading causes of death in the world, among which, oral cancer is associated with significant morbidity, and low survival. A large part of the budget allocated to health care is attributed to cancer. In this study we aim to estimate the economic burden of oral cancer in Iran for the year 2014.

**Methods:**

In this study, we generated a prevalence-based estimate of the cost-of-illness of oral cancer in Iran. A societal perspective was used for this study, in which the direct costs and productivity losses of oral cancer cases in 2014 were estimated. The human capital approach was adopted for estimating productivity losses. Several data sources contributed to this study, including national cancer registry reports, hospital records, occupational data, and interviews with experts.

**Result:**

Nearly 53% of patients were diagnosed in an advanced stage of oral cancer. The economic burden of oral cancer was $64,245,173 most of which (50%) was attributed to productivity losses. The direct medical cost accounted for 42% of the estimated total cost. Treatment expenses for advanced stages were five times higher than the early stages ($10,532 vs. $2,225).

**Conclusion:**

The economic burden of oral cancer is high in Iran. Planning an early detection and screening program for oral cancer may potentially decrease health care costs, morbidity, and mortality.

## Introduction

Oral cancer is the sixth most prevalent cancer type across the world. This type involves 2–3 percent of all cancer cases [1]. With an annual rate of 400,000 new patients afflicted with oral cancer and 130,000 deaths, this cancer is one the most significant causes of death, especially in developing countries [2]. This cancer includes various types of malignant neoplasms in the oral cavity such as tumors of lips, gum, mouth floor, soft palate, hard palate, the tonsils and salivary glands. Epidemiological studies show that the prevalence of oral cancer varies geographically from 0.1 to over 40 percent in different parts of the world [3]. Based on the latest reports, the oral cancer incidence rate in Iran is about 1400 new cases per 100,000 individuals per year, and the age standardized incidence rate (ASR) was 2.2 for men and 1.8 for women [4]. In countries such as India, Pakistan, Bangladesh and Sri Lanka, the mentioned cancer type has been more prevalent in men rising to consist 25 percent of the total number of all cases [1, 5]. Oral cancer is more common in men compared to women [3, 6]. The proportion of oral and throat cancer in men to women is 1.5 to 1 and 2.8 to 1, respectively. However, this proportion is decreasing in the last decades due to less tobacco use and elevation of life expectancies in women [1].

Cancer is the second cause of mortality in Iran [7]. The mortality number of oral cancer in Iran is about 450 new cases per year, with age standardized mortality rates (ASMR) of 0.7 in men and 0.6 in women per 100,000 people [4]. Also, oral cancer is among the top ten cancer types in several provinces of Iran [8].

Aside from considerable effects on the patients’ health and their quality of life, oral cancer imposes costs on households and also on the health system. Through describing the cost-of-illness, a much more comprehensive understanding of the importance of health problems can be acquired [9]. Awareness on the amount of expenses of a disease can help policy makers decide on prioritizing health care, prevention, and treatment policies. Besides, such estimations can help policy makers evaluate the efficiency of different strategies in the health sector [10–12].

The purpose of measuring the economic burden of disease is to provide the most realistic evidence needed for policy-making, designing and managing health plans, prioritizing strategic investigations about population health, developing and dedicating human and financial resources, and enhancing the capacities of organizations in designing, implementing, and assessing cost-effective interventions of prevention, treatment, and rehabilitation [11, 13, 14]. Despite the important role the economic burden of cancer cases plays for decision makers in allocating budget to important programs in Iran, there are only a few studies conducted in this area [15–18]. Hence, this is an attempt to provide a prevalence-based estimate of all costs associated with oral cancer from a societal perspective in Iran in 2014.

## Materials and methods

‘Prevalence-based' and ‘incidence-based’ methods are the two main approaches used for estimating the economic burden of diseases. Incidence-based studies estimate lifetime costs of a disease since its emergence until the time of being healed or the time of death, and are attributed with an appropriate discounting rate to the year the disease was diagnosed for the first time. Prevalence-based studies measure disease-attributable costs that occur concurrently with prevalent cases over a specified time period, usually 1 year. This approach is considered most suitable for assessing the total current economic burden of a health problem [19]. We adopted the prevalence-based approach to estimate the economic burden imposed by oral cancer in the year 2014. This study was conducted from a social perspective. The costs included in the analysis were direct medical, direct non-medical, and indirect costs.

### Estimating the prevalence of oral cancer in Iran

Since more than 90% of oral cancer patients pass away within five years after diagnosis, we used the five-year prevalence as the overall prevalence of oral cancer in 2014 (20, 21). We used the GLOBOCAN 2012 database provided by the International Agency for Research on Cancer (IARC) to estimate the overall prevalence of oral cancer. Since the GLOBOCAN data are not age specific, we used data from Iran National Cancer Registry Report published by Ministry of Health in Iran to determine its distribution in different age groups. We then adjusted the prevalence rate reported by GLOBOCAN with these data [8, 22–24]. Data were collected from patients who were in-patients or out-patients in the Cancer institute, Imam Khomeini Hospital, Tehran, Iran. It is the largest referral center for cancer patients in Iran (under the supervision of Tehran University of Medical Sciences), with at least 10,000 various Cancerous Subjects annually from all over the country. Demographic data, pathologic data for staging cancer and direct medical costs were obtained from the patient records. Data for direct non-medical and indirect costs were collected through telephone interviews with the patient or his family members. A structured questionnaire was used to collect information S1 File. The same survey question was adapted that was used in previous studies [25, 26]. In order to access the patients' records, the necessary authorization was obtained from the Deputy Director of Research and Technology of the Iran University of Medical Sciences (Ethics Committee Code: IR.IUMS.REC 1395.9211552205). Also, the goals of research are described in the telephone interview and the interviewees’ verbal consent was gained.

### Direct medical costs

We extracted diagnostic and treatment process from the National Comprehensive Cancer Network (NCCN) guideline in order to estimate direct costs of oral cancer based on the early (stages I and II) and advanced (III and IV) stages. Then we standardized the NCCN process by the expert panel for Iran. The expert panel included medical oncologist, radiation oncologist, Head and Neck surgeon and pathologist. Diagnostic measures include expert visits, biopsy, MRI, CT scan, and dental examinations. Patients diagnosed in early stages (I and II) of the cancer receive surgery or radiotherapy in the first line of treatment. After that, if the patient has adverse features (according to experts in 15% of patients) he/she should receive re-treatment. In the second line of treatment, patients who have been previously undergone surgery, receive radiotherapy and patients who have received radiotherapy in the first line of treatment, undergo surgery.

Patients in advanced stages (III and IV) receive surgery, and then according to patient conditions and pathology of cancer, they receive radiotherapy or undergo chemotherapy and radiotherapy simultaneously. According to experts, recurrence occurs in 10% of the patient in early stages (I or II) and 40% of ones in advanced stages (III and IV). After each treatment, patients are followed up to assess the recovery process or recurrence of cancer. Follow-up measures include expert visits (three times a year), MRI (every six months) and CT scan (every six months). The average cost of each diagnostic and therapeutic measure in different stages of the disease was extracted from patient records. Finally, the average cost was determined per patient [27]. We used expert opinions to standardize the practice in Iran’s Medical tariff of 2014, published by the ministry of health for both public and private sectors to estimate the services costs. [28]

### Direct non-medical costs

The transportation and home care costs were estimated considering the lack of studies and data about the non-medical costs of cancer patients, including oral cancer patients. A questionnaire was utilized to calculate the direct non-medical costs. Data were obtained by a telephone interview with the patient or with one of his or her family members.

### Indirect costs

Indirect costs of oral cancer represent loss of productivity due to disability, absence from work and premature death. The human capital approach was adopted to estimate the indirect costs. The productivity losses associated with morbidity and mortality are the ‘market value’ of that individual’s future contribution to production in a society if s/he had continued to work in full health. The human capital approach assumes that the monetary value of productivity lost due to morbidity or premature death caused by an illness equals the current wage. Therefore, the active population (15 to 65 years) was considered in order to estimate the lost productivity [14, 29]. The earnings in future years are discounted using appropriate rates [12]. To estimate the lost productivity cost due to disability, first patients and their families were interviewed to calculate the number of days that each patient lost due to oral cancer in 2014. Then, the average number of lost days was multiplied by the average daily wage. Different daily wages were used for employed and unemployed patients. For unemployed patients, the minimum daily wage approved by the Ministry of Cooperatives Labour and Social Welfare of Iran in 2014 was considered [30]. Usually a family member accompanies the patient during visits; thus, the time costs for a family member as the patient were estimated, assuming that the family members are unemployed, and so the minimum wage rate was applied for them.

To determine the cost of productivity lost due to premature death in oral cancer, first the number of deaths due to oral cancer was estimated and categorized based on age groups and gender using the data available in the IARC (GLOBOCAN) and the Ministry of health [8, 24]. Secondly, the number of years lost in each age group was derived from subtraction of the median age group from the 2014 life expectancy rate, as reported in the World Health Organization database [31]. Finally, the lost years in each age group were estimated by multiplying the number of deaths in each age group with the corresponding life expectancy. The minimal annual wage and the annual average wage was adopted for unemployed, and employed patients, respectively. Data on employment rates in each age group by gender, average annual wage and minimum annual wage were obtained from the Ministry of Cooperation, Labor and Social Welfare of Iran in 2014 [30]. A 5% discount rate was considered to convert the stream of lifetime earnings into a present value. All costs were reported in US dollars using the average annual 2014 exchange rate (US$1 = Rial 32,510).

## Results

In total, 114 patient records with complete pathological and financial information were included in the study. Most patients were male (62.2%), with an average age of 55.27 ± 3.42. More than half of the patients (59.6%) had been diagnosed in advanced stages, and most cases of cancer were squamous cell carcinoma. The tongue was the most common sites of tumor origin. The 5-year prevalence and mortality of oral cancer were estimated 3024 and 449 respectively in Iran in 2014. Tumor staging of studied patients was based on TNM staging system, the most widely used Method to classify the local extension of the primary tumor (T), locoregional lymph node involvement (N), and the presence of metastasis (M). Distribution of tumor location, tumor staging and the prevalence rate is shown in Table 1.

**Table 1:** Studied patient characteristics and epidemiological data of oral cancer 2014.

Characteristics of cancer patient	Number(percent)
**Gender**	
Male	71 (62.2)
Female	43 (37.8)
**Site of cancer**	
Floor of Mouth	23 (20.1)
Lip	17 (14.9)
Tongue	63 (55.2)
Buccal	11 (9.6)
**Type of cancer**	
Squamous Cell Carcinoma	96 (84.2)
Other	18 (15.7)
**Stage**	
Early (I, II)	53 (46.5)
Advanced (III, IV)	61 (53.5)
**Epidemiology Data**	
Incidence	1380
1 year prevalence	827
3 year prevalence	2057
5 year prevalence	3024
Mortality	449

The components of the direct medical costs of oral cancer are presented in Table 2. Since diagnostic and follow-up procedures were performed for almost all patients, the expenditure on these procedures was calculated for all cases. The cost of surgery in advanced stages of cancer was about three times higher than the one in the early stages ($1,243 vs. $3,020). Also, the cost of radiotherapy in advanced stages of cancer was nearly three times higher than the same measure in early ones ($1,051 vs. $3,152). The cost of disease treatment (including surgery, radiotherapy and chemotherapy) in stages III and IV makes up 70% of direct medical costs.

**Table 2:** The direct costs of oral cancer management in Iran in 2014.

Cost type	Number of patients	Direct medical costs		
		Per patient	Total	percentage
Diagnosis	1380	82	113,160	0.5
Stages I and II (surgery and radiotherapy)	1,210	2,225	2,691,027	9.9
Stages III and IV(surgery, radiotherapy and chemotherapy	1,814	10,532	19,108,982	70
Recurrent	847	1,485	4,491,941	16.5
Follow-up	3,024	291	879,391	3.1
Total cost			27,284,501	100

The direct non-medical costs of oral cancer in 2014 are presented in Table 3. According to a telephone interview, almost all patients require care during or after being discharged from the hospital. The average home care days were 94 days. In more than 90% of cases, patients care was conducted by a spouse or family member, whereas in less than 10% care was provided by a trained nurse, nurse or practical nurse. Also the average transportation for patients consisted of approximately 17 trips. The average cost of traveling and caring for patients at home were $1,035.4 and $665.5 respectively.

**Table 3:** The direct non-medical costs due to oral cancer in Iran in 2014.

Cost type		Travelling costs	Home care costs	Total
Direct Non-medical costs	Per patient	1,035.4	665.5	1,700.9
	Total	3,131,157	2,012,472	5,143,629

The average days of disability and absence from work were 174 and 68 days for patients and their accompanying individuals, respectively. Since the mean monthly wage for every employed and unemployed person was $427 and $212, respectively, we estimated that the total cost of disability and absence from work was the approximate amount of $4,933,797 in 2014 (Table 4).

**Table 4:** The indirect costs of disability and absence from work due to oral cancer in Iran in 2014.

status	Number	Mean of missed work days	Mean Cost per patient, $US	Total cost, $US
Employed patients	1,352	174	2,477	3,348,517
Unemployed patients	547	174	1,230	672,622
Accompanies	1,899	68	481	912,658
Total				4,933,797

Our findings showed that of the total number of 449 deaths from oral cancer, 253 cases were between the ages of 15 and 65, and the other 196 were for people over 65. The average lost years due to premature death of oral cancer per patient was 24 years. The highest number of deaths occurred in the age group of 50–54 years and 55–59 years old. Also, the highest mortality cost was for the age group of 54–55 ($ 5,403,844). The average productivity loss was $3612 per year and $14,449 per patient. The lost productivity costs due to premature mortality of oral cancer in 2014 are presented in Table 5.

**Table 5:** The indirect costs of oral cancer due to premature mortality in Iran in 2014.

Age group (year)	Number of death		Number of years lost	Mean mortality cost	Total mortality cost
	men	women			
15–19	1	0	58	254,402	254,402
20–24	3	2	265	232,471	1,162,353
25–29	2	1	144	210,539	631,618
30–34	8	3	473	188,608	2,074,690
35–39	5	4	342	166,677	1,500,093
40–44	17	8	825	144,746	3,618,645
45–49	31	13	1,232	122,815	5,403,844
50–54	39	16	1,265	100,883	5,548,590
55–59	26	19	810	78,952	3,552,852
60–64	34	21	715	57,021	3,136,159
Total	166	87	6,129		26,883,246

**Fig 1:**
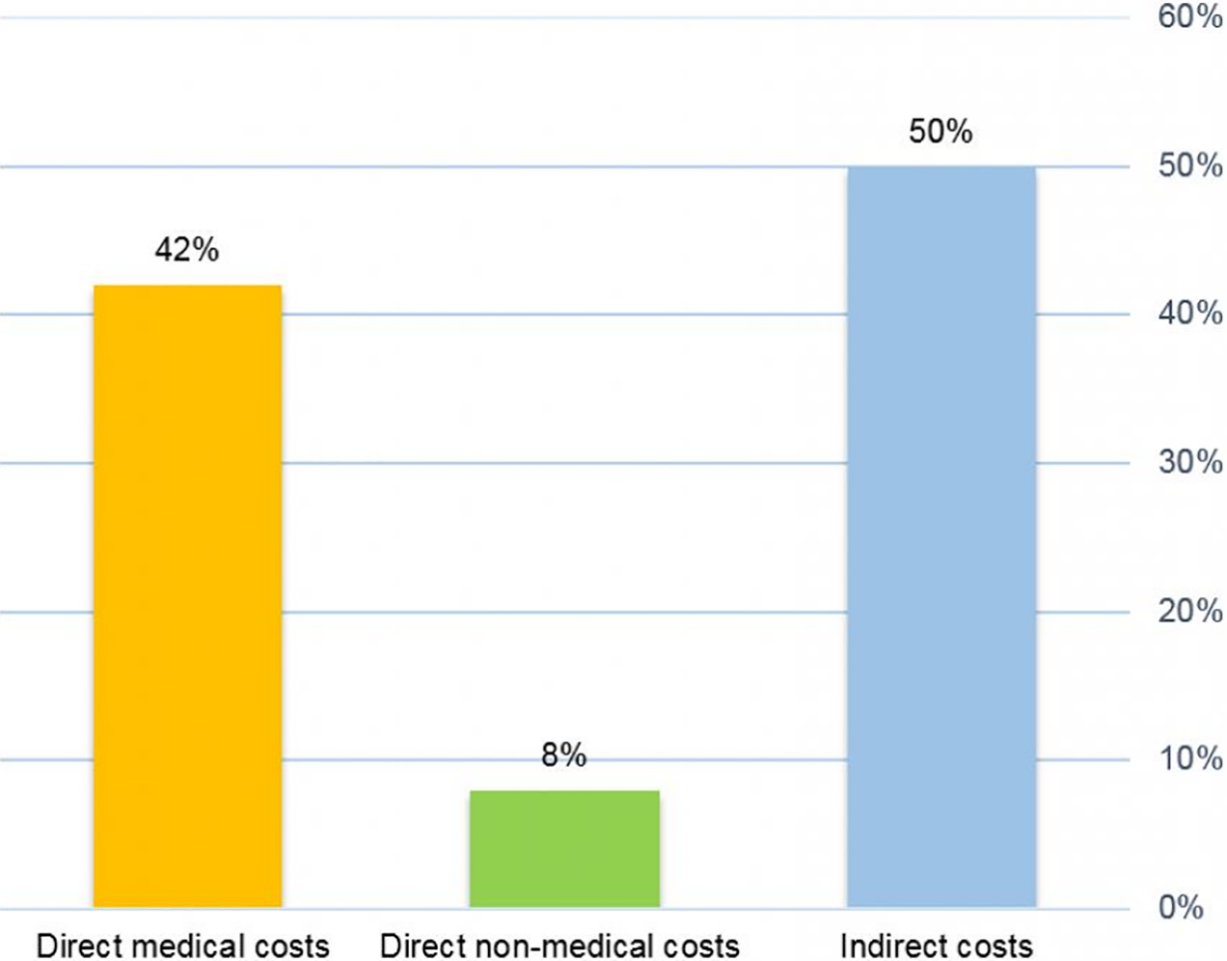
The economic burden of oral cancer in Iran in 2014.

Iran’s economic burden of oral cancer in 2014 was $64,245,173. The main components of the cost were mortality and direct medical cost (Fig 1).

## Discussion

The present study is the first attempt conducted to quantify the economic burden of oral cancer incurred to Iran. The present study on the cost-of-illness used a prevalence-based, human capital approach to estimate such annual costs. Most of the identified patients were diagnosed with an advanced stage of cancer. The cost of treatment in stages ІІІ and ІV consisted a major part of direct medical cost. It is indicated here that Iran's economic burden of oral cancer in 2014 amounted to $31,817,043 and most of this cost was attributed to lost productivity.

The findings of the present study demonstrate that 47% of the attended patients were in the early stages, and 53% were in the advanced stages of cancer. Esmaeil Beigi et al. (2014) examined 206 oral cancer patients, showing that 71.4% patients were in the advanced stages of the disease, while in study of Sargeran and et al. (2008), which included 470 oral cancer patients in a hospital in Tehran, more than 58% of patients were found to be in such stages. [20, 21]. Also there are several studies in other countries reporting different portions of patients with advanced disease stages (Stage III or IV), ranging from 57% to 75% [32–34].

Despite the screening simplicity of oral cancer, less than half of such cases are detected in an early stage. On one hand, the main reason for delay in detection is that the disease is asymptomatic and without pain in the early stages. On the other hand, adults’ level of awareness concerning the risk factors and symptoms of oral cancer is low. A study in Iran suggested that 50% of people are aware of the causes of oral cancer, and only 30% are aware of their symptoms [35]. Moreover, dentists' knowledge about oral cancer is not sufficient in Iran [36–39].

The findings showed that the average treatment cost of oral cancer is $9,022 per patient. Furthermore, the average treatment costs for early and advanced stages of the disease were $2,225 and $10,532, respectively. Our study indicated that the average cost of surgery in the advanced stage was more than two times higher than the cost of surgery in the early stage. Also, the costs of cancer treatment in the first year for the advanced stages (III and IV) were approximately three times higher than in the early stages (I and II). In a study by Dedhia et al (2011) in the United States, the cost of hospitalization for oral cancer, in advanced stages, was more than two times higher than in the early stages ($24,842 vs. $10,101) [40]. The study of Speight et al (2006) showed the total cost of treatment over a period of three years in the first, second, third and fourth stages was 4914, 8534, 11.883, and 13.513 pounds respectively [41]. The results of our study were confirmed by other studies [32, 42, 43].

Earlier diagnosis of oral cancer increases the likelihood of treatment with a single modality, lowers the risk of mortality, decreases medical expenditures, and improves patients’ quality of life [32]. In the advanced stages of cancer, due to the deterioration of patients' conditions, large tumors and metastasis, patients received a combination of different treatments. However, in the early stages of cancer, most patients received only one type of treatment. The number of hospitalization days, complications and costs related to the treatment of those patients who are in advanced stages of cancer are greater.

The lost productivity of oral cancer in Iran in 2014 was approximately $32 million, most of which (62%) due to premature death in men. In a study by Pierce et al (2015) in Ireland the major share of costs was related to premature mortality [44]. In Western Europe, lip and oral cavity cancer is ranked seventh with lost production of €400,000 in men and more than €150,000 in women [45]. In the United States, lost productivity is nearly $2.8 billion per year. [46]. In Germany, the cost of absence from work due to oral cancer was €17.5 million [47]. Other studies also found that the number of deaths from oral cancer in men is larger than the one in women [1, 48, 49]. One of the reasons for this is the risk factors of these cancers; tobacco and alcohol, which are more commonly used by men than women. Indirect costs of cancer are due to the absence of temporary or permanent work, disability, reduced working hours or early death. In some studies, only one or some of these cases are considered as indirect costs, and in some studies, it includes all cases. This confirms the differences observed in the mentioned studies. Also, the incidence and prevalence of cancer and its mortality are variable from country to country.

The average mortality rate of oral cancer in the world is about 20%, WHO Regional Office for the Eastern Mediterranean (EMRO)22.5%, and 14% per year in Iran. Although Iran's average is lower than the one of the world's and the one of EMRO’s, the average mortality rate in the United States, United Kingdom and Germany is 5.4, 8.9, and 8.6 percent, respectively [24]. This difference can be due to proper screening and identification of patients in the early stages of cancer. Moreover, another reason can be the advanced technology and treatment methods in these countries. A randomized control trial on screening for oral cancer conducted in India has demonstrated a significant mortality reduction in tobacco users [50]. Also, Dedhia et al. suggested that the annual oral cancer screening for high-risk American males over 40 years of age could be considered cost-effective. This figure is derived from both savings in costs of management ($258) and increase in quality-adjusted life years ($3105) of the screen group [40].

Despite public education, the proportion of patients presenting with advanced-staged diseases have not changed in the last 40 years. [1]. It is well established that the treatment of the early stages oral cancers achieves higher survival rates with less attendant morbidity and that at present far too many patients present with advanced stage disease.

Policy makers' interventions are necessary to reduce the oral cancer incidence and mortality. Therefore, screening for premalignant or early stages of oral cancer cases is worthy of consideration. Having a well-developed prioritization of available resources to disseminate enough awareness about oral cancer among population and improve having equal access to care is proposed by WHO and the Breast Health Global Initiative (BHCI) as basic suggestions in dealing with oral cancer in countries with low and middle income. Thus, for exercising a preventive strategy in controlling oral cancer, this study aimed to propose a comprehensive plan which is built upon two key points, first, the health providers’ training priorities, especially for oral practitioner like dentists and oral hygienists, and second, early detection and diagnostic of cancer. As a preliminary but critical phase, this study also presents a plan for providing cost-effectiveness evaluation data prior to the national screening program. Broadly speaking, the output of this study will inform the next steps and studies associated with cost-effectiveness estimation of the disease burden with and without screening prior to starting a nationwide program in each community.

This is the first study which estimated the economic burden of oral cancer in terms of early and advanced stages in Iran, several data sources, including national cancer registry reports, hospital records, occupational data, and interviews with experts. Furthermore, this study has some limitations. First, due to the lack of accurate information on the prevalence and mortality of oral cancer in Iran and its calculation infeasibility, the data were obtained from GLOBOCAN 2012 and Ministry of Health in Iran, which are apparently underestimated [51, 52]. Second, the treatment tariff of the Ministry of Health in Iran was used here, which may not reflect the real cost; therefore, our results could underestimate the economic burden of these diseases. Third, we used the human capital approach to calculate the indirect cost. This method tends to underestimate costs because it values life using market earnings, thereby yielding very low values for children and the retired elderly. In addition, psychosocial costs, such as pain and suffering, are components of the burden of illness omitted from the computation of indirect costs of human capital. We believe that these results should be interpreted with caution. And finally, confirmation by larger, standardized, prospective studies is needed.

## Conclusion

The economic burden of oral cancer in Iran seems to be considerable. The great extent is related to productivity lost due to premature death. The results of this study provide useful information for health care providers and decision makers in understanding the economic burden of oral cancer. Hence, the policy makers are recommended to adopt screening in oral cancer as a highly effective strategy in treatment and management of this disease.

## Supporting information

S1 File (DOCX)
