# Supplementary material for: The economic burden of oral cancer in Iran
Source: PLoS One. 2018 Sep 27;13(9):e0203059. doi: 10.1371/journal.pone.0203059 (PMC6160006; doi:10.1371/journal.pone.0203059)
Supplement: S1 File — (DOCX) [file pone.0203059.s001.docx]

**Economic burden of oral cancer questionnaire**

Personal Information:

1- Sex: Male □ Female □

2. Marital status: Single□ married □ Divorced □ Widowed □

3- Age: .............. years

4. Duration of the disease (since it was diagnosed): .................. months .......... years

5. Education level: illiterate □ fifth elementary school □ less than diploma □ diploma □

  Associate Degree□ Bachelor □ Masters Degree □ Doctorate degree □

6. Occupation: Employee □ Military □ Self-employed □ Farmer □ Soldier □

A homemaker □ A student □ Retired □ Unemployed □

Please list other: ...........................

8. Average Household Income: .................................

Less than 500 000 tomans □ 500 000 – 1 000 000 tomans □ 1 000 000 – 2 000 000 tomans □ 2 000 000 – 3 000 000 tomans □ 3000 000- 4 000 000 tomans □ More than 4000000 tomans □

9) Which province and city do you live in? Province: ...................... city: ........................ ...

10. Where is your place of residence? City □ Village □

12. Are you a household head: Yes □ No □

13. Are you covered by insurance? Yes □ No □

14. If you are covered by insurance, specify your type of insurance?

Compulsory Social Security □ Voluntary Social Security □

Government staff insurance □ Iranian insurance □ Villagers insurance □

Military insurance □ Medical services and insurance (Imam Khomeini Relief Committee) □

If you are covered by another insurance cover: .............................

15. Are you covered by supplementary insurance? Yes □ No □

16. If you have a supplementary insurance, specify the type: ………………………

**B) treatment costs**

7. Did you leave your city or province for healthcare services last year? Yes □ No □

8. If the answer to the above question is yes then answer the following questions:

How many times have you left your city last year (for treatment)?

............. times

• Which of the following items do you use?

Airplan □ Bus □ Train □ Private car □ Intercity Taxi □

 • List other items: .....................

• On average, how long was your trip every time? ................ of the day

• If you used to stay in Tehran or other cities in the last year to receive treatment, which of the following do you use to stay?

Personal home □ family relatives □ The inn □ hotel □ public places □

List other things: ..........................

• If you have paid for your stay, please indicate the average cost per stay: ................ Tomans

• Do you have someone accompany you to go to Tehran or other cities? Yes □ No □

• What is the average cost per travel time? (If you have a companion, add the cost to him/her) ................................. Tomans

9. Which of the following are usually used for your Intra-city travel for treatment?

Bus □ Metro □ Taxi □ Motor □ Other Equipment □

10. How much was the cost of Intra-city travel each time? .............. Tomans

11. On average, How many hours (days) do you need each time you visit a doctor and buy medicine?

1 hour □ 2-3 hours □ 5-3 hours □ 7-5 hours □ 10-7 hours □

12. Do you need to accompany for an outpatient visit to a doctor?

always □ most of the time □ rarely □ don't need any time □

13. Have you bought medications during and after your hospitalization to your current illness in the Last year?

Yes □ No □

14. If the answer to the above question is yes, how much was the cost of buying this drug or drugs?

....................

15. Have you been forced to take sick leave or absenteeism over the last year due to your current illness? Yes □ No □

16. If the answer is yes, specify the date: ........... days

17. Have you had nursing and home care during the last year? Yes □ No □

18. If the answer is yes, mention it and indicate the type of caregiver.

Duration of home care: ............ days

Caregiver type:

Trained nurse □ Experimental urse □ Paramedic □

Spouse □ children □ other relatives □

19. If you have paid for home care, please include: ........................ Tomans

**پرسشنامه بار بیماری سرطان دهان**

« باسمه تعالی»

**الف) مشخصات فردی:**

1- جنسیت: مرد□ زن□ 2- وضعیت تأهل: مجرد□ متاهل□ مطلقه□ همسر فوت کرده□

3- سن: .............. سال

4- مدت ابتلا به بیماری (از زمان تشخیص آن) : ........... ماه .......... سال

5- میزان تحصیلات: بی سواد□ پنجم ابتدایی□ کمتر از دیپلم□ دیپلم □

فوق دیپلم□ لیسانس□ فوق لیسانس □ دکتری □

6- شغل: کارمند□ نظامی□ آزاد□ کشاورز□ سرباز□

خانه دار□ محصّل □ بازنشسته□ بیکار□ سایر موارد را ذکر نمایید: ...........................

8- متوسط درآمد خانوار: …………………………… کمتر از 500 هزارتومان□ 1000000 - 500 هزارتومان□ 1500000 – 1000000 تومان □ 1500000 تا یک 2000000 تومان□ بیشتر از 2000000 تومان□

9- ساکن کدام استان و شهرستان هستید؟ استان: ...................... شهرستان: ...........................

10- محل زندگی شما کجاست؟ شهر□ روستا□

12- آیا سرپرست خانوار می باشید: بلی□ خیر□

13- ؟ بلی□ خیر□

14- در صورتی که تحت پوشش بیمه می باشید نوع بیمه خود را مشخص نمایید؟

تامین اجتماعی اجباری □ تامین اجتماعی اختیاری□

کارکنان دولت □ خدمات اجتماعی □

بیمه ایرانیان □ بیمه روستاییان □

بیمه نیروهای مسلح □ بیمه کمیته امداد □

سایر اقشار □

اگر تحت پوشش بیمه دیگری می باشید آنرا ذکر نمایید: .............................

15- آیا تحت پوشش بیمه تکمیلی درمان هستید؟ بلی□ خیر□

16- در صورتی که بیمه تکمیلی دارید نوع آن را ذکر نمایید: ................................................

**ب) هزینه های درمان**

7- آیا در سال گذشته برای دریافت خدمات درمانی از شهر یا استان خود خارج شده اید؟ بلی□ خیر□

8- در صورتی که جواب سوال فوق بلی می باشد به سوالات زیر پاسخ دهید:

در سال گذشته چندمرتبه از شهر خود (جهت دریافت درمان) خارج شده اید؟ ............. مرتبه

- معمولااز کدام یک از وسایل زیر استفاده نموده اید؟ هواپیما□ اتوبوس□ قطار□ اتومبیل شخصی□ تاکسی بین شهری□
- سایر موارد را ذکر نمایید: .....................
- به طور متوسط هر بار مسافرت شما چه مدت به طول انجامیده است ؟................ روز
- در صورتی که در سال گذشته برای دریافت خدمات درمانی نیازمند اقامت در تهران یا سایر شهرها بوده اید از کدام یک از موارد زیر جهت اقامت استفاده نموده اید؟ منزل شخصی□ منزل اقوام □ مسافرخانه□ هتل□ مکان های عمومی مثل مسجد، حسینیه و... □

سایر موارد را ذکر نمایید: ..........................

- در صورتی که برای اقامت خویش هزینه ای پرداخته اید متوسط هزینه هربار اقامت خود را ذکر نمایید: ............... هزار تومان
- آیا برای مراجعه به تهران یا سایر شهرها همراه داشته اید؟ بلی□ خیر□
- به طور متوسط هزینه هر بار مسافرت شما چقدر بوده است(در صورت داشتن همراه هزینه وی را هم اضافه نمایید) ؟ ................................. هزار تومان

9- معمولا برای حمل ونقل داخل شهری خود جهت دریافت خدمات درمانی از کدامیک از وسایل زیر استفاده می نمایید؟

اتوبوس□ مترو□ تاکسی□ موتور□ آژانس□ سایر وسایل□

10- به طور متوسط هزینه هر بار رفت و برگشت داخل شهری خود را جهت دریافت خدمات درمانی چقدر می دانید؟ .............. هزار تومان

11- به طور متوسط برای هر بار ویزیت پزشک وخرید دارو به چند ساعت (روز) وقت نیاز دارید؟

1ساعت□ 3-2 ساعت□ 5-3 ساعت□ 7-5 ساعت□ 10-7 ساعت□

12- آیا برای مراجعه سرپایی به پزشک همراه دارید؟

همیشه همراه دارم □ بیشتر اوقات همراه دارم□

به ندرت همراه دارم □ هیچ گاه همراه ندارم□

13- آیا درسال گذشته در حین بستری و بعد از آن برای درمان بیماری فعلی خود به خرید دارو اقدام نموده اید؟

بلی□ خیر □

14- اگر جواب سوال فوق بلی می باشد، هزینه خرید این دارو یا داروها چقدر بوده است؟ ....................

15- آیا در طی سال گذشته به خاطر بیماری کنونی خود مجبور به اخذ مرخصی استعلاجی، یا غیبت از کار شده اید؟ بلی□ خیر□

16- در صورتی که جواب بلی است مدت آنرا ذکر نمایید: ........... روز

17- آیا در طی سال گذشته نیاز به پرستاری و مراقبت در منزل داشته اید؟ بلی□ خیر□

18- در صورتی که جواب بلی است مدت آن را ذکر نمایید و نیز نوع مراقب را مشخص نمایید.

مدت مراقبت انجام شده در منزل: ............ روز

نوع مراقب: پرستار آموزش دیده □ پرستار تجربی□ بهیار□

همسر □ فرزندان□ سایر اقوام□

19- اگر هزینه ای بابت پرستاری در منزل پرداخته اید مقدار آنرا ذکر نمایید: ........................ هزار تومان
